# Whole-genome nanopore sequencing and automatic downstream analysis of respiratory syncytial virus using RSVTyper

**DOI:** 10.1038/s41598-025-20371-5

**Published:** 2025-10-31

**Authors:** Duyen Bao Le, Inga Tometten, Nadine Lübke, Martha Paluschinski, Anna-Kathrin Schupp, Lutz Ehlkes, Pascal Kreuzer, Nicole Zacharias, Jörg Timm, Alexander Dilthey, Andreas Walker

**Affiliations:** 1 Institute of Virology , University Hospital Düsseldorf Heinrich Heine University Düsseldorf , Düsseldorf, Germany; 2 Düsseldorf Health Authority (Gesundheitsamt Düsseldorf) , Düsseldorf, Germany; 3 Institute for Hygiene and Public Health , University Hospital Bonn , Bonn, Germany; 4 Institute of Medical Microbiology and Hospital Hygiene , University Hospital Düsseldorf Heinrich Heine University Düsseldorf , Düsseldorf, Germany; 5 Center for Digital Medicine , Heinrich Heine University Düsseldorf , Düsseldorf, Germany

**Keywords:** Biological techniques, Biotechnology, Computational biology and bioinformatics, Microbiology, Molecular biology

## Abstract

Respiratory syncytial virus (RSV) is a globally circulating virus, causing severe respiratory infections in infants and the elderly. Two RSV vaccines were recently approved, and passive immunization is now recommended in several countries for all newborns, therefore careful surveillance of RSV variants will be important in the future. We therefore develop an integrated whole genome RSV amplification, sequencing and bioinformatics analysis method („RSVTyper“; https://anaconda.org/bioconda/rsv-typer) that is suitable for patient samples as well as wastewater. 243 RSV isolates from 2008 to 2025 and wastewater samples from 2023/2024 were amplified in a multiplex tiling PCR with specific primers, generating 39 amplicons ~ 550 bp in length, and sequenced with Oxford Nanopore Technologies. Sequencing reads of patient isolates were analyzed with the RSVTyper pipeline, a tailored analysis pipeline including automatic reference selection, consensus sequence generation and clade assignment via Nextclade. Amplification and sequencing were successful for 213/243 samples. Whole genomes (> 90% coverage) were obtained from 98% of samples with > 10,000 copies/mL, from 3/14 samples with 1,000–10,000 copies/mL, and from none with < 1,000 copies/mL. Average genome-wide mean depth for successfully sequenced samples was 31,800x with an average mean depth of 41,400x in the F gene. Phylogenetic analysis showed seasonal clade and subtype shifting, with a good representation of clade frequencies from patients in wastewater sequences. No variants with known escape mutations from prophylactic monoclonal antibodies were detected. In conclusion, we developed RSVTyper, a cost-effective and scalable RSV sequencing pipeline by integrating sequencing and bioinformatic analysis. It is suitable for both resource-limited settings and high-throughput applications. It will facilitate enhanced RSV surveillance, allowing for further characterization of the RSV genome and rapid detection of potential escape mutations.

**Supplementary Information:**

The online version contains supplementary material available at 10.1038/s41598-025-20371-5.

## Introduction

Respiratory syncytial virus (RSV) is a globally prevalent virus that primarily infects the respiratory tract, causing symptoms ranging from mild to severe illness^1^. RSV can lead to serious lower respiratory tract infection, particularly in infants and the elderly. The virus is highly contagious, with reinfections commonly occurring throughout lifetime^2^. Due to its high transmissibility, RSV spreads rapidly in communal settings such as daycare centers and hospitals, leading to significant seasonal outbreaks in pediatric care. Preterm infants and children with underlying pulmonary or cardiac health conditions are especially vulnerable, with elevated morbidity and mortality risks^3^, especially in low and middle incoming countries^4^.

Human RSV isolates are classified into two antigenic groups, RSV-A and RSV-B, which diverged approximately 350 years ago^1^. Within these groups, there are multiple subgenotypes, initially defined based on the sequence of the second hypervariable region of the highly variable G gene^5^. In 1998, a 60-nt duplication was identified in the G gene of RSV-B that rapidly spread and became the dominant strain worldwide^6^. Similarly, in RSV-A a 72-nt duplication in the G gene appeared 2012^7^ that also replaced the circulating prototype and became dominant. Multiple subtypes can circulate simultaneously within the population, with dominant subtypes typically shifting from year to year. The distribution of genotypes often follows a one- to two-year cycle, largely explained by existing population partial immunity^8^. However, reinfections with the same genotype can also occur.

Until recently, prophylactic administration of Palivizumab (Synagis™; MedImmune), a neutralizing F-protein-specific monoclonal antibody, was the standard to protect newborns at risk from severe RSV infection^9^. However, due to its relatively short half-life, this treatment must be administered intramuscularly on a monthly basis throughout the entire RSV season. With the recently approved monoclonal antibody Nirsevimab^10–12^, a neutralizing antibody with improved stability and longer half-life is available and is now recommended in several countries for single-dose prophylaxis at the start of the RSV season for all newborn children, regardless of comorbidities^13,14^. In addition, two RSV vaccines for active immunization, based on the F protein locked in the prefusion conformation, in adults were recently approved^15^.

The COVID-19 pandemic highlighted the importance of early detection and monitoring of circulating viral sequence variants to understand subtype evolution^16,17^ and identify resistance as it emerges^18,19^. Currently, numerous different RSV sequencing protocols^20–22^, mostly based on short-read data, have been published. Here, we developed a short amplicon RSV sequencing protocol using nanopore sequencing that is effective for both patient samples as well as wastewater surveillance.

## Results

### Design of a multiplex primer set for efficient amplification of RSV

**Fig. 1:**
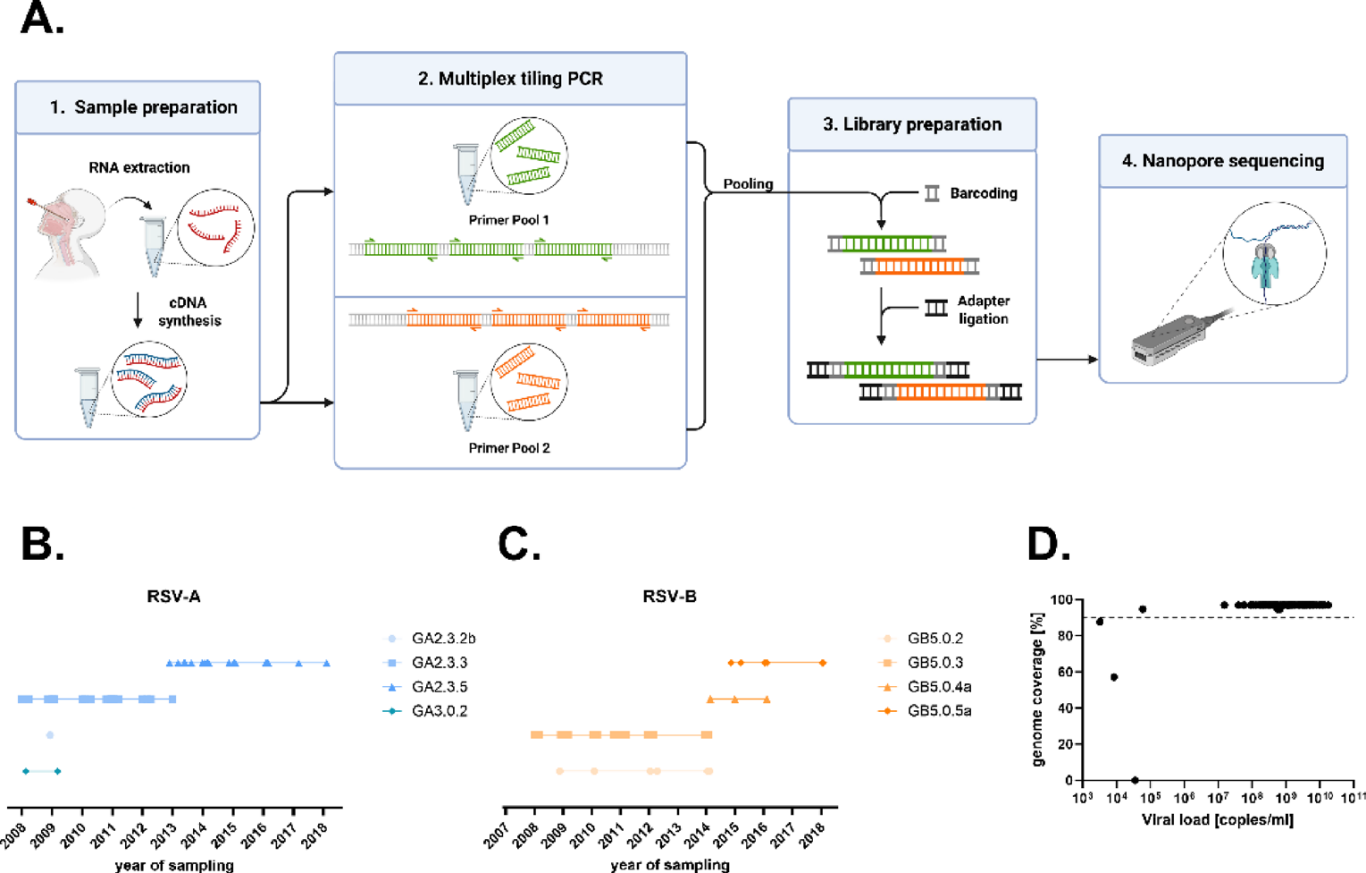
Overview of the RSVTyper pipeline. (**A**) Wetlab workflow. First, RNA was extracted and transcribed into cDNA, which was then amplified in a multiplex tiling PCR in two primer pools. The PCR products were pooled and libraries were prepared for nanopore sequencing. (**B**, **C**) RSV cell culture isolates and their G clades are depicted for each sampling year, spanning from 2008 to 2018. (**D**) Genome coverage of each sample is depicted in relation to its viral load. Genome coverages were calculated from the consensus sequences generated by the RSVTyper pipeline. Whole genome sequences were defined as sequences with a genome coverage > 90%. The dashed line indicates 90% genome coverage.

Different strategies for amplification of RSV are available, including large amplicon protocols^20^, hybrid capture^21^ or short amplicon combined with Illumina sequencing^22^. While these methods perform well with high-quality samples and high viral loads, they are more prone to amplicon dropout when RNA quality or quantity is low. To enable RSV sequencing with nanopore sequencing also from challenging materials like wastewater, an RSV pan-subtype amplicon scheme was designed for amplification of PCR fragments with a length of approximately 500 bp. In brief, we designed 126 RSV-specific primers (65 in pool 1 and 61 in pool 2), generating 39 overlapping amplicons (Suppl. Figure 2 and Suppl. Table 1, RSV-Primer). A schematic representation of the overview is shown in Fig. 1A.

To analyze the suitability of the primer set, 95 RSV isolates from the years 2008–2018 (49 A and 46 B), each passaged once in cell culture, (Fig. 1B,C) were used. The median DNA concentration after amplification was 117 ng/µL (range: 19.3-220.3 ng/µL) and all samples were nanopore sequenced generating a median of 420 megabases (range 28–737) per isolate. Each sample yielded on average 573,108 (range 58,662-1,022,623) reads before and 496,911 (range 35,192–895,604) reads after the length filter, resulting in a whole genome coverage (mean depth) of 14,602x (range 1,898–26,908) and a F gene coverage of 19,143 × (1,243–31,324) (for individual values see Suppl. Table 2). Using a minimum read depth of 20 unique reads per position the average genome coverage was 95% (Fig. 1D and Suppl. Table 2). Successfully sequenced samples were defined as > 90% of the genome resolved. Samples with < 90% genome coverage were classified as failed. In total, 92 of 95 cell culture isolates were successfully amplified and sequenced using the RSVTyper primer scheme. The remaining three had viral loads < 40,000 copies/mL (Fig. 1D and Suppl. Table 2).

### Development of a bioinformatics pipeline for the generation of RSV consensus sequences

**Fig. 2:**
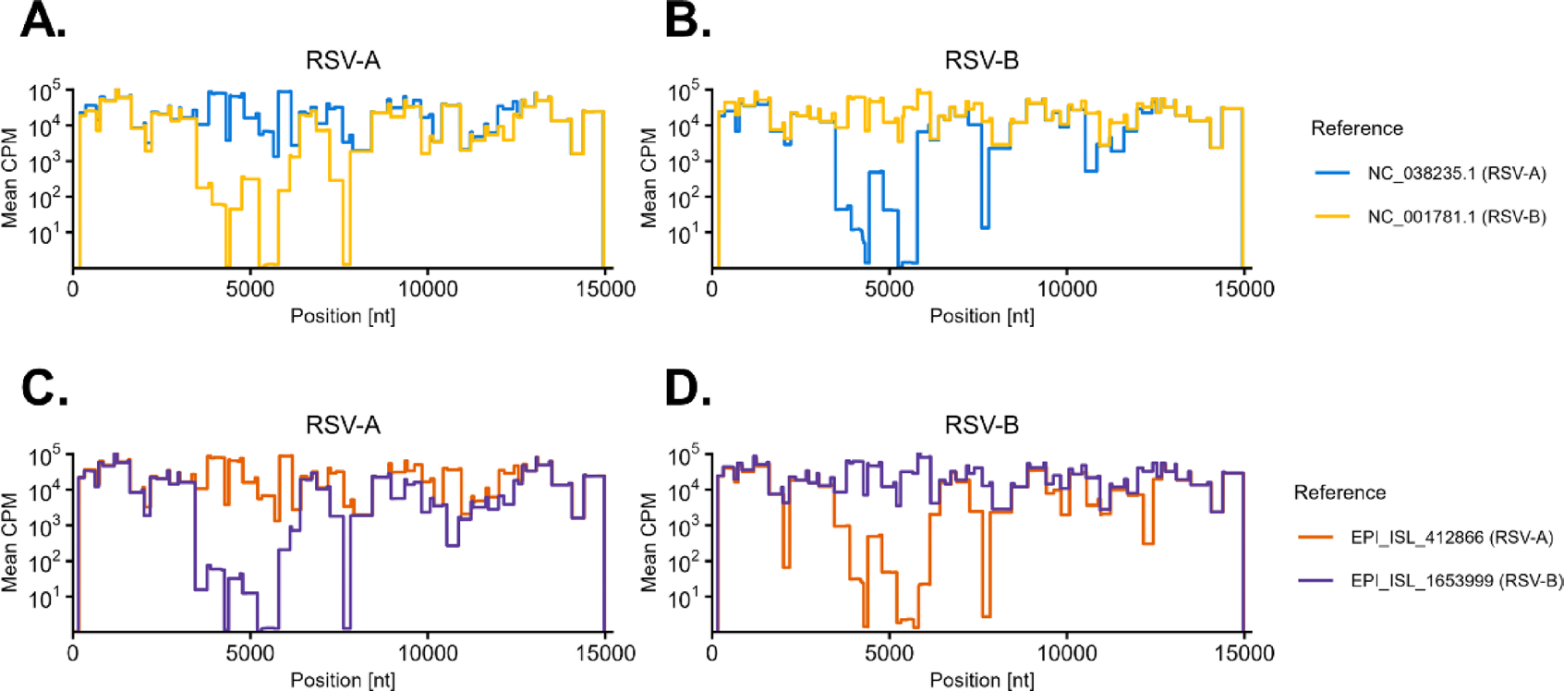
Effect of reference sequence selection for read mapping. Reads from cell culture isolates were mapped to different reference sequences with the ARTIC pipeline. (**A**) RSV-A isolates mapped against NCBI reference sequences (**B**) RSV-B isolates mapped against NCBI reference sequences (**C**) RSV-A isolates mapped against GISAID reference sequences (**D**) RSV-B isolates mapped against GISAID reference sequences. Average counts per million (CPM) mapped reads are depicted for each position.

The field bioinformatics pipeline provided by the ARTIC Network is a ready to use data processing pipeline for Oxford Nanopore Technologies (ONT) sequencing data (https://community.artic.network/). Demultiplexed and polished ONT reads are aligned to the reference genome, producing a consensus sequence, a list of detected variants and a BAM file for visualization. Specific protocols are provided for SARS-CoV2^23^ and Ebola^24^, but not for highly diverse viruses with multiple genotypes. During sequencing of the cell culture isolates, it became evident that the use of an incorrect RSV subtype as a reference in the ARTIC pipeline significantly reduced read mapping efficiency. To systematically assess the impact of the reference sequence on mapping performance, we re-analyzed all cell culture isolates using representative reference genomes from both NCBI and GISAID. Using an RSV-B reference for RSV-A isolates resulted in a significant loss of mapped reads (Fig. 2A–C). This was particularly problematic in regions between nt 4,200-5,800 and nt 7,600-7,800, where the mapping frequency dropped from 50,000x to < 10x or from 2,000x to 0, when the wrong reference was used, respectively. The same problem occurred when RSV-B isolates were mapped to an RSV-A reference (Fig. 2B–D). Inconsistent results were observed with old reference sequences from NCBI (Fig. 2A,B) as well as newer ones from GISAID (Fig. 2C,D), indicating that usage of the wrong reference sequence leads to read alignment coverage dropouts. To avoid the use of a false reference, an automatic RSV subtype detection as a first step before implementation of the ARTIC pipeline was introduced. In this step, the RSVTyper aligns all reads against a panel of RSV-A and RSV-B sequences and counts the number of reads aligning to the highly diverse region between 3,500 and 5,500 nt of the reference. The subtype of the sequence with the highest read count is then chosen for further analysis.

**Fig. 3:**
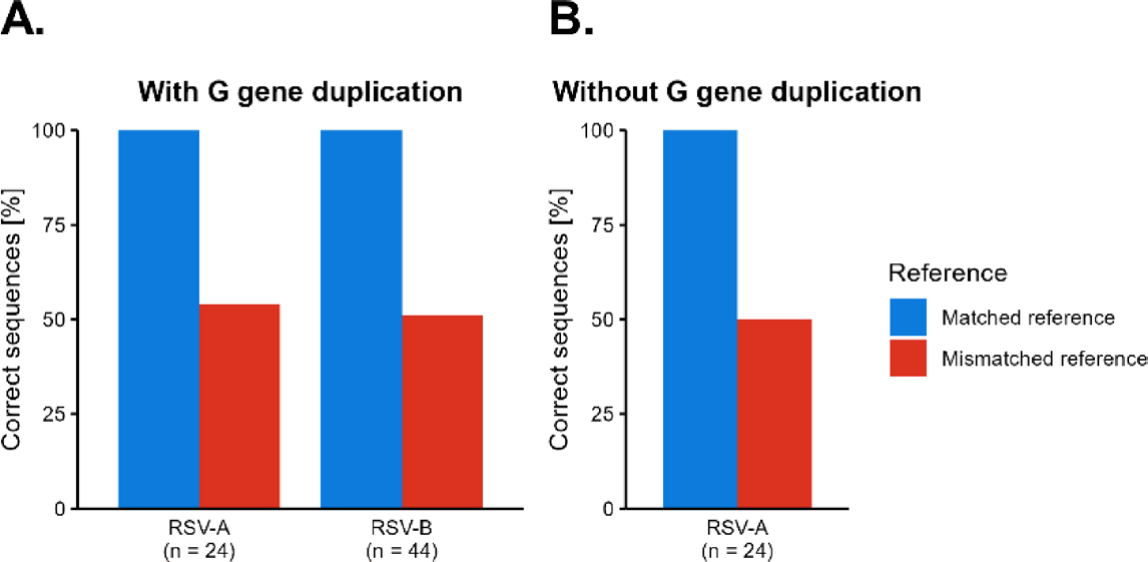
Effect of G gene duplication on consensus generation by medaka. The percentage of samples with a correctly generated G gene sequence is depicted depending on the selected reference in the ARTIC pipeline. All samples were mapped to a reference with or without the G gene duplication. Matched reference refers to using references that correspond to the presence or absence of the G gene duplication in the sequenced isolates. (**A**) RSV-A and RSV-B isolates with the G gene duplication. Matched references contained the G gene duplication, mismatched references did not. (**B**) RSV-A isolates without the G gene duplication. Matched references did not contain the G gene duplication, mismatched references did. RSV-B isolates without the G gene duplication were not available.

The algorithms in the ARTIC pipeline used for consensus sequence generation sometimes ignore duplications when they are not in the reference sequence. Therefore, the effect on consensus sequence generation in presence or absence of the G gene duplication was analyzed. Mapping RSV reads from isolates containing the G gene duplication to RSV references lacking the G gene duplication resulted in an incorrect G gene sequence in 54% of RSV-A and 51% of RSV-B isolates. Variants ranged from ignored G gene duplication, partially called G gene duplication to G genes with small deletions (Fig. 3A, red bar). A similar pattern was observed for RSV isolates lacking the G gene duplication when mapped to an RSV reference containing G gene duplication. Here, only RSV-A strains lacking the G gene duplication were available and again consensus sequences of the G gene were incorrectly generated for 48% of the isolates (Fig. 3B).

To prevent incorrect G gene calling, an additional reference selection step was added into the workflow, choosing either a reference containing or lacking the G gene duplication.

As we observed differences in generated consensus sequences depending on the reference used, we decided to create our own reference panel consisting of 10 RSV-A and 10 RSV-B references. This reference panel is a diverse set of RSV sequences based on sequences from GISAID and NCBI, enabling analysis of RSV strains from a wide time range. To generate the most accurate consensus sequence, a final reference selection step that detects the most suitable reference under consideration of previously called subtype and G gene structure was added.

**Fig. 4:**
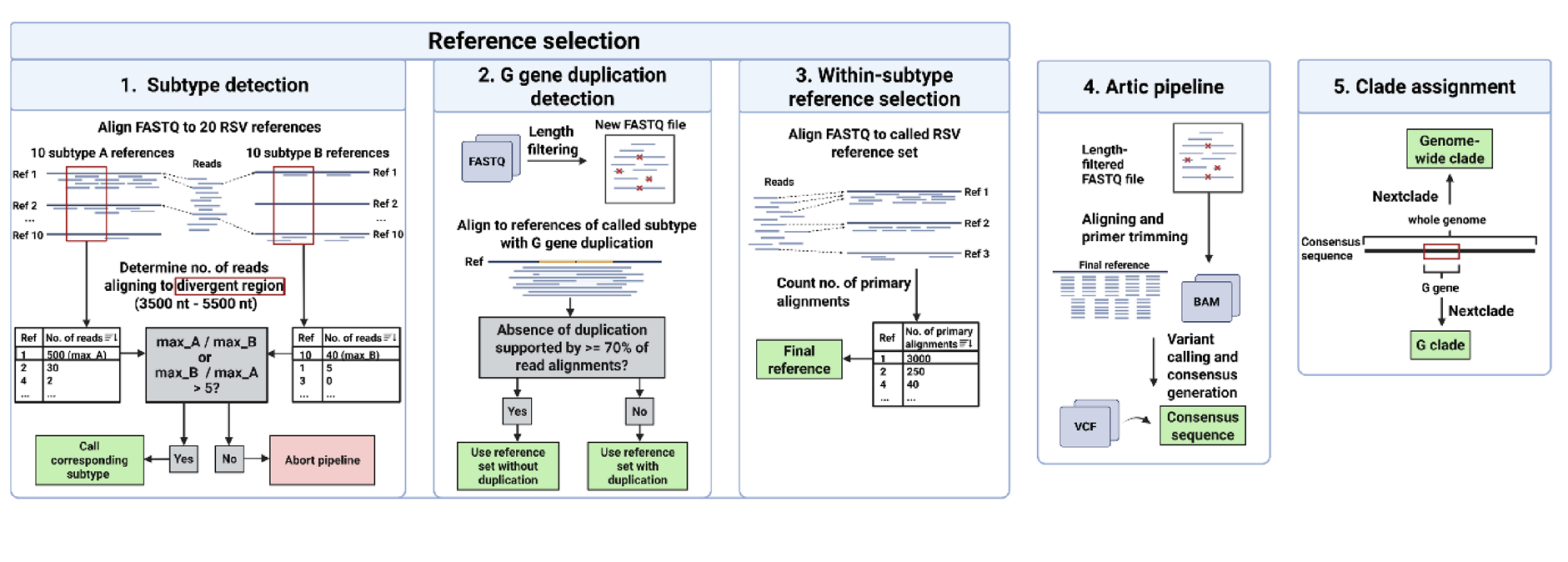
Computational workflow. The first three steps were performed to select the reference which resembles the isolate the most. The subtype and the presence of the G gene duplication were first detected. Then, from a subset of references with corresponding features, the final reference was selected. Consensus sequences were then generated using the ARTIC pipeline and G clade and genome-wide clades were called using Nextclade.

This optimized workflow was implemented in a user friendly Bioconda package that is freely available (https://anaconda.org/bioconda/rsv-typer and https://github.com/DiltheyLab/RSVTyper). (Fig. 4).

**Fig. 5:**
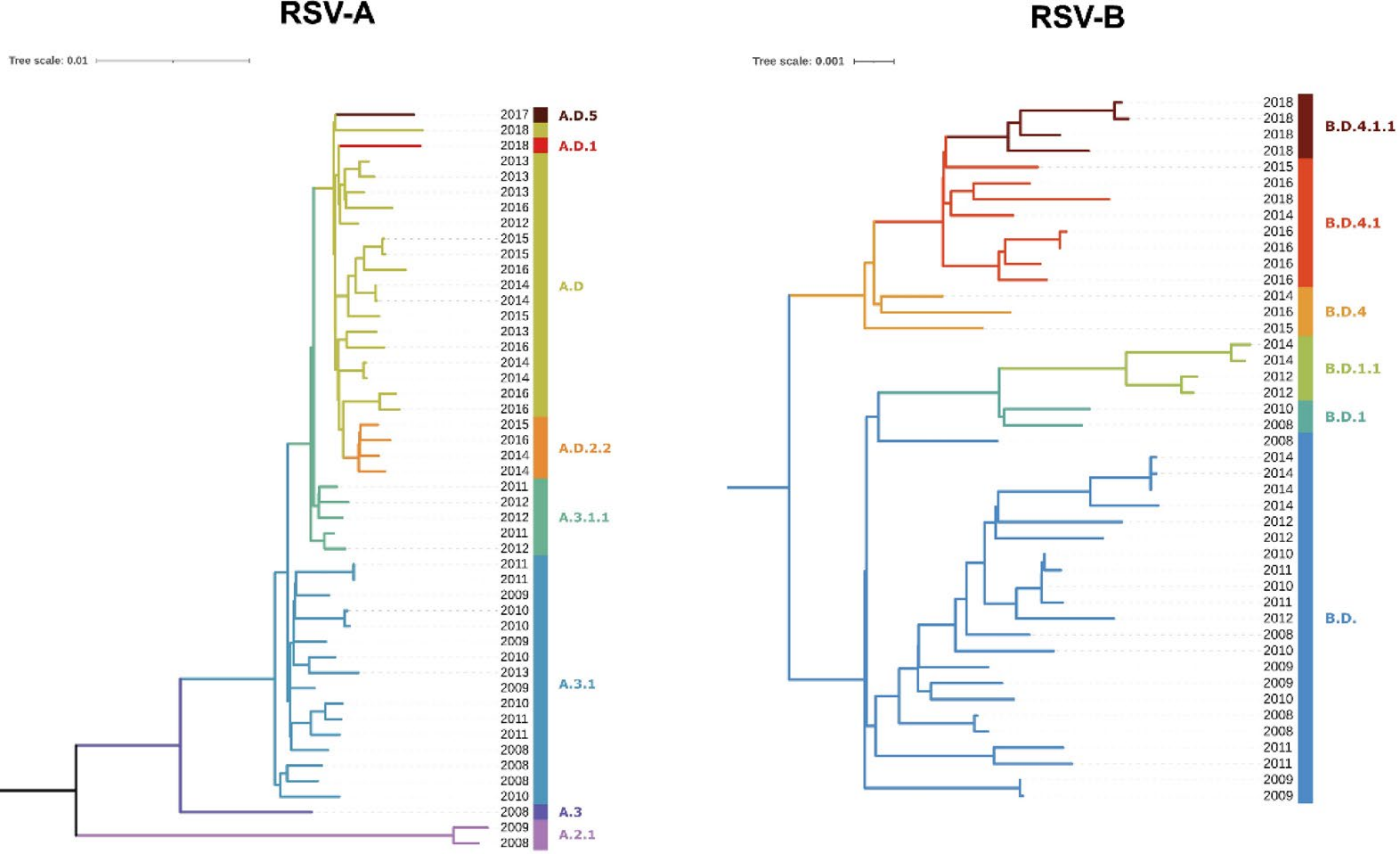
Emergence of new variants over time. Whole genome sequences generated by the RSVTyper from RSV cell culture isolates for RSV-A (left, *n* = 48) and RSV-B (right, *n* = 44) were aligned using MAFFT and Neighbor-joining trees were computed with MEGA using the Tamura-Nei model and visualized using iTOL v7. The sampling year of each isolate is denoted at the end of each branch. Branches are colored by assigned genome-wide clades (as implemented in Nextclade) which are displayed next to the sampling year.

All cell culture isolates were analyzed again using the optimized computational workflow. To compare clade assignment as implemented on the website Nextclade (https://clades.nextstrain.org^25^ to phylogeny, phylogenetic trees were calculated and the clade IDs, defined by the RSV Genotyping Consortium^26^, were assigned. The tree and the years from which the isolates originate are shown in Fig. 5. There was a clear correlation between the year of isolation and the corresponding Nextclade reference for RSV-A (left) and RSV-B (right) isolates, demonstrating that the consensus sequences generated by the RSVTyper are consistent with the isolates’ year of origin.

### Efficient amplification and typing of clinical isolates between 2023 and 2025

**Fig. 6:**
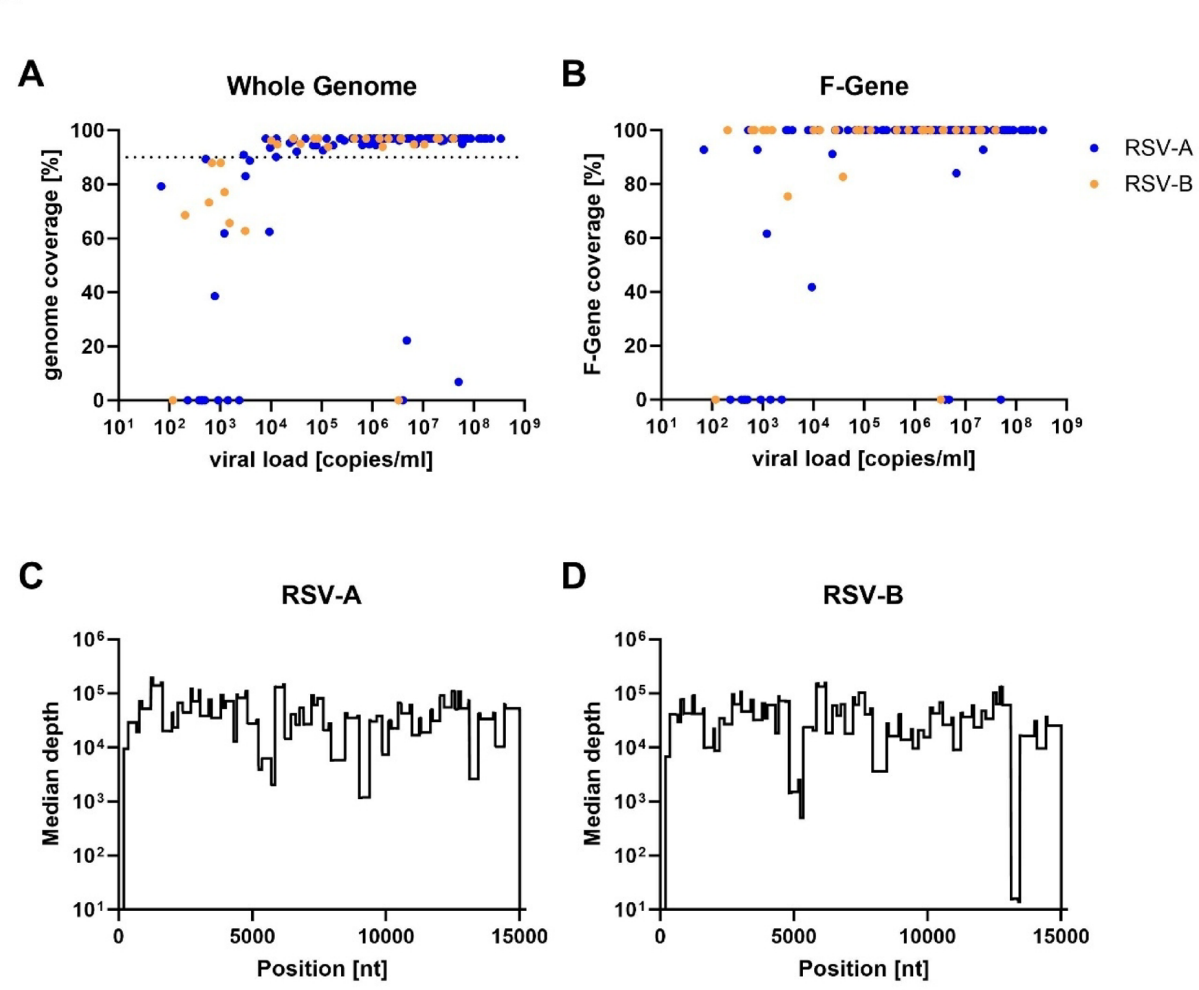
Successful amplification of clinical isolates. (A + B) Genome coverage of sequenced clinical isolates in relation to their viral load (*n* = 148). RSV-A isolates are colored in blue and RSV-B isolates in orange. (**A**) Genome coverage of the complete genome. The dashed line indicates 90% genome coverage. (**B**) Coverage of the F-Gene coding sequence (1725nt). (B + C) Median sequencing depth across the RSV genome for (**B**) RSV-A (*n* = 112) and (**C**) RSV-B (*n* = 28) isolates.

Following the establishment of the RSVTyper, nasopharyngeal swabs from clinical samples collected at the University Hospital Düsseldorf during RSV seasons 2023/24 and 2024/25 were analyzed (for details see Suppl. Table 2). The cohort comprised 120 RSV-A and 28 RSV-B isolates, with a median viral load of 1.8 × 10^7^ copies/mL (range: 69 − 3.3 × 10^8^ copies/mL). Complete genome sequences were successfully resolved from 121 out of 148 samples (82%), including 102 of 120 RSV-A isolates (85%) and 19 of 28 RSV-B isolates (68%). A clear correlation between viral loads and genome coverage was observed. Complete genomes (coverages > 90%) were resolved from 118 of 122 (97%) samples with a viral load of > 10,000 copies/mL (Fig. 6A). Only three complete genomes were recovered from samples with < 10,000 copies/mL, all of which had viral loads > 1,000 copies/mL. Of note, 8 of the 28 RSV-B isolates had viral load < 10,000 copies/ml (Fig. 6A, orange dots). The F gene serves as the primary neutralizing target for monoclonal antibodies and is a key component of all licensed vaccines. Consequently, establishing a robust amplification scheme for this region is of critical importance. Complete F gene coverage was achieved in 102 out of 120 RSV-A isolates (85%) and 24 out of 28 RSV-B isolates (85%), even in samples with less than 90% total genome coverage (Fig. 6B). For RSV-A, the median sequencing read depth exceeded 1,000x across the entire genome (Fig. 6C). Similar results were observed for RSV-B, except for two genomic regions, corresponding to the G and L gene, which showed slightly reduced coverage (Fig. 6D).

**Fig. 7:**
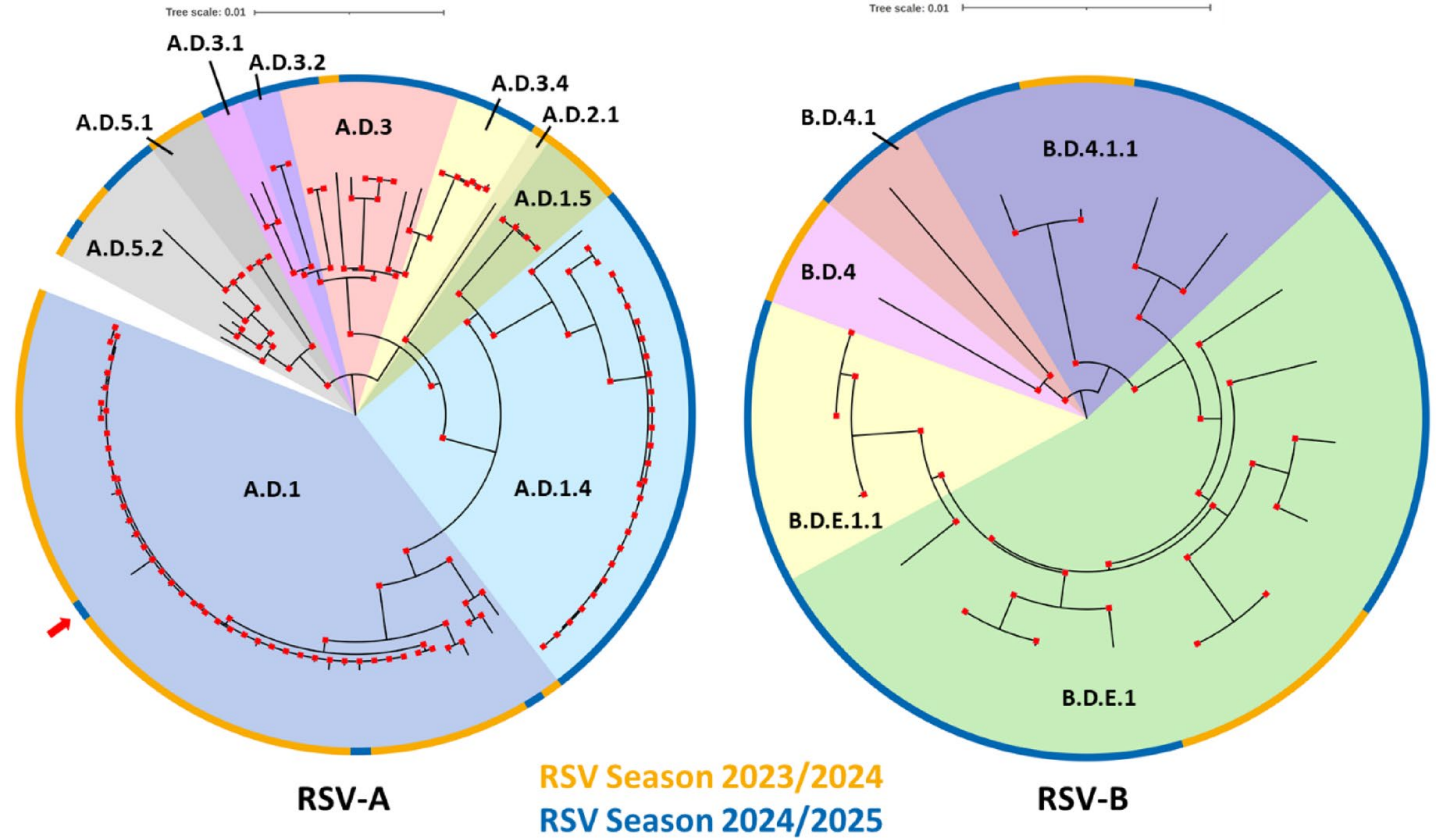
Phylogenetic analysis of RSV isolates from clinical samples from the University Hospital Düsseldorf, RSV seasons 2023/2024 and 2024/2025.

Next, the clades defined by the RSV Genotyping Consortium^26^, derived by the built-in Nextclade clade assignment tool in the RSVTyper, were compared to a phylogenetic tree inferred by a Bayesian approach (Fig. 7). Clade assignment and phylogenetic placement correlated very well and nearly all nodes had a posterior probability of 1. All RSV-B isolates were classified to the B.D.4 lineage and its sublineages (Fig. 7 right). The evolutionary development of sublineages from B.D.4.1.1 could also be clearly observed: the B.D.E.1 lineage (also known as B.D.4.1.1.1) branches off from B.D.4.1.1, and B.D.E.1.1.1 further branches off from B.D.E.1. In contrast, RSV-A isolates exhibited greater genetic diversity. While some RSV-A sequences were assigned to the A.D.5 lineages, the majority of samples from season 2023/24 were classified within the A.D.1 clade. Despite the majority of A.D.1 isolates originating from 2023/24, there were also three isolates from 2024/25. Interestingly, one isolate from January 2025 (HHU-177, marked with a red arrow in Fig. 7) showed a significantly higher genetic distance compared to the other 2023/24 isolates. Relative to the consensus sequence of the other A.D.1 isolates, it contained 19 evenly distributed synonymous mutations and two non-synonymous mutations located in the M2 and L coding regions. Recombination analysis (Suppl. Figure 3) revealed no evidence of recombination or notable differences, suggesting either strong genetic drift or an introduction from abroad as reason for the high genetic distance. In the 2024/25 season the dominating RSV clade is A.D.1.4. Additionally, several RSV-A strains belonged to A.D.3 and its sublineages. Analysis of the protein sequences revealed that none of the sequenced isolates carried known escape mutations against nirsevimab (Suppl. Table 3).

Whole genome sequences of 102 RSV-A (left) and 19 RSV-B (right) were aligned using MAFFT and a phylogenetic tree was calculated using Mr.Bayes. The consensus tree represents the maximum clade credibility tree, with posterior probability values shown at the nodes as a measure of branch support. Nodes with posterior probability 1 are shown by red squares. Assigned genome-wide clades derived from Nextclade and are highlighted by different background colors within the trees. Samples originating from RSV season 2023/2024 and RSV season 2024/2025 are denoted by yellow and blue color strips at the end of each branch, respectively. The isolate from season 2024/2025, belonging to the A.D.1 clade is marked by a red arrow.

### The RSVTyper multiplex approach allows amplification and sequencing of RSV from wastewater

**Fig. 8:**
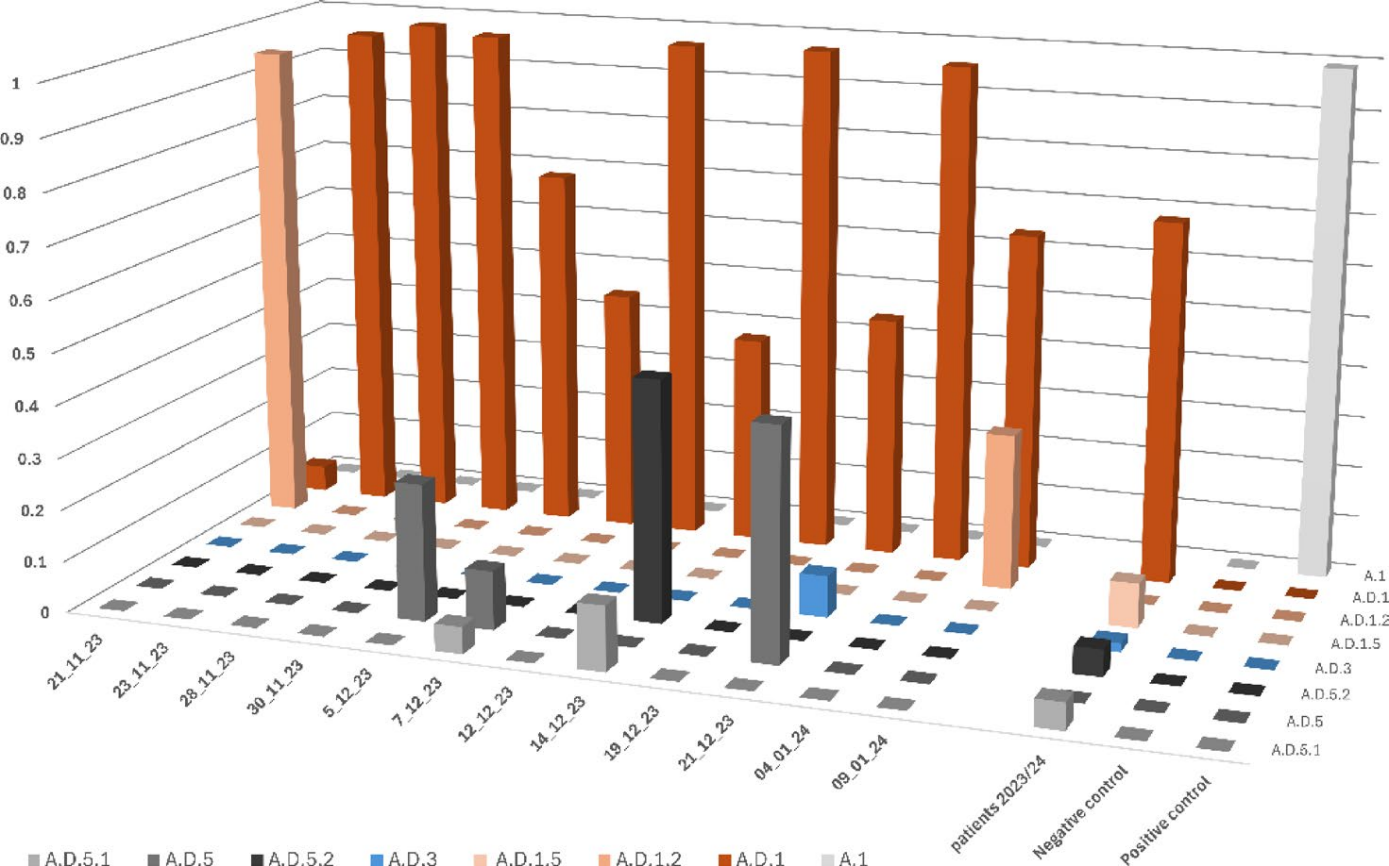
RSV-A variant abundances in wastewater from RSV season 2023/2024. RSV-A (A) variants detected in wastewater samples from 12 different timepoints are compared to RSV-A (*n* = 54) variants detected in patients from the University Hospital Düsseldorf, sampled within the same time frame, respectively.

Wastewater analysis is a valuable tool for epidemiological monitoring, enabling rapid detection of pathogens and screening for viral variants. To assess RSV amplification in wastewater, RNA was extracted from wastewater samples collected in Düsseldorf, Germany, over a three-month period during the 2023/24 season. The RNA was then amplified using our RSV-specific primer set and sequenced with ONT. Although PCR yields were much lower (13–23 ng/µL) compared to patient samples, we could obtain 65,747 reads/per sample on average (range 10,634 − 180,922). Frequency of the individual RSV variants was analyzed using the variant detection algorithm Freyja^27^. It uses lineage-defining mutations to estimate relative variant abundances in mixed samples. Variants with frequencies > 0.05 are shown in Fig. 8 and were compared to isolates found in patients from the same time frame. As expected, the most frequent RSV strain found in patients, A.D.1, was also reproducibly found in wastewater (Fig. 8). Also, isolates A.D.5.1 and A.D.5.2, less frequently detected in patients, could be found at several time points in the wastewater. Read count for RSV-B were much lower than for RSV-A, which is in line with the low number of RSV-B infections in Düsseldorf during that season. Due to the low read count, the detection was less precise and in most samples the variants could only be resolved down to the parent variant B.D.4.1 (Suppl. Figure 1).

## Discussion

In addition to a newly approved passive immunization, the first active vaccines against RSV have been approved in the past two years. The simultaneous implementation of multiple immunization strategies including a broad use of monoclonal neutralizing antibodies in all newborns will most likely increase selection pressure on the virus. Therefore, increased surveillance of RSV variants will be important in the future. The RSVTyper presented in this study offers a complete package to sequence RSV with an amplicon-based tiling approach using ONT. Besides the RSV-A and B specific primer scheme, the RSVTyper also includes a ready-to-use bioinformatic pipeline available through Bioconda. High quality genomes were generated from cell culture and patient isolates and variants were successfully detected in wastewater.

The RSVTyper method shows robust amplification efficiency and the sequencing depth generated by nanopore sequencing is sufficient for high genome coverage. The usage of RSV-A and RSV-B pan-subtype primers improve the handling performance, compared to subtype-specific protocols^22^. Hybridization capture techniques^21^ are less sensitive to sequence variations but are more expensive to implement, more time-consuming, and require a high degree of automation to enable high throughput. In contrast, amplicon-based methods are inexpensive to implement and can be performed with standard laboratory equipment. Compared to large 1.5 kb-4 kb amplicon-based RSV sequencing schemes^20,28,29^, smaller amplicons are more robust regarding low RNA quality or low quantity^24^. In line with this, complete genomes in patient samples with more than 1,000 copies/mL and RSV sequences from wastewater could be obtained. While our overall efficiency appeared lower compared to other studies, this is largely attributable to our inclusive approach, as we did not exclude low viral load samples (< 10,000 copies/mL; ~Ct > 31)^20,21,29^. Notably, applying a higher cutoff at 10,000 copies/mL would have yielded complete genomes for 210 out of 215 samples (98%), demonstrating the robustness of our method even under more stringent conditions. The success rate of obtaining complete genomes from patient isolates seems lower for RSV-B than RSV-A, likely due to 8 of the 28 RSV-B samples having viral loads < 10,000 copies/mL. Additionally, the observed amplicon dropout in the L gene of RSV-B was not caused by poor primer binding, as analysis of the primer binding sites revealed no mismatches in these isolates. Comparable dropouts have also been observed by others^30^, suggesting that additional factors may also be responsible. Although unfavorable secondary RNA structures could play a role, the limited number of RSV-B isolates (*n* = 28) from the 2023–2024 season prevents us from drawing definitive conclusions. Importantly, the F gene—the primary target of current active and passive immunization strategies—was fully resolved in almost all isolates. Notably, F-gene completeness did not differ between RSV-A and RSV-B, indicating that our method is well suited for detecting potential vaccine escape mutants. As outlined by Goya et al.^31^ recently, Pneumovirus genome misassemblies, in this case the G gene duplication, has been a persisting issue due to misalignments to references and it is suggested that approx. 4% of RSV-sequences in the databases isolates after 2022 are misassembled e.g. do not contain the G-gene duplication. Our approach circumvents this issue in two ways: experimentally, we have designed primers which generate amplicons spanning the G gene duplication. Additionally, due to the usage of ONT, there is no fragment size limit. We can therefore sequence the amplicon potentially carrying the G gene duplication as a whole and do not encounter misalignments to overlapping regions as observed in short-read sequencing. Bioinformatically, we have implemented a G gene duplication detection step to determine which reference reflects the sample the most. When mapping reads of a sample without the G gene duplication, this is visible in the alignment as deletions at that region. A sample with the G gene duplication would fully map to the region. We make use of this difference in the alignment to determine the most-suited reference.

Sequencing of patient isolates is essential for RSV surveillance; however, wastewater surveillance offers additional advantages. It covers the entire population of a city or district, rather than only including ill patients who seek medical care. Consequently, wastewater surveillance enables unbiased screening for variants of concern, including early detection in a population^32^. Similarly, RSV surveillance in wastewater may help identify vaccine breakthroughs at an early stage. The RSVTyper multiplex approach was successfully used to amplify RSV from wastewater samples. Furthermore, the frequency of variants detected in wastewater samples was comparable to that found in patient samples from University Hospital Düsseldorf, suggesting that wastewater surveillance is representative of circulating RSV isolates. Consistent with the low number of RSV infections in patients, RSV-B was also less detected in wastewater, limiting precise variant detection.

More than 90% of RSV related childhood mortality occur in low- and middle-income countries (LMICs), however only 14.8% of publicly available RSV sequences were from patients recruited in LMICs^33^. Here, our workflow offers additional opportunities. Amplicon sequencing only requires a PCR cycler and cheap laboratory equipment. Nanopore sequencing requires no costly equipment, can be performed in small research laboratories or resource-limited settings and allows rapid analysis during sequencing^16^. Finally, by selecting the appropriate flow cell, anywhere from a few to 96 samples can be sequenced simultaneously. Of note, the amplicons generated by the RSVTyper wetlab protocol can also be submitted to Illumina sequencing, in case higher throughput is required.

The key advantage of the RSVTyper pipeline is the integration of the wetlab nanopore sequencing protocol into a comprehensive bioinformatics workflow that can be easily installed via Bioconda. This enables researchers with limited computational or software experience to perform RSV sequencing and analysis using a standardized approach. Given the limited data on RSV circulation in low- and middle-income countries, the development of methods that can be implemented with limited resources and minimal bioinformatics expertise is crucial from a global health perspective. Additionally, amplification with small PCR fragments facilitates the analysis of challenging sample types, such as wastewater, opening new possibilities for RSV surveillance.

## Materials and methods

### Patient and wastewater samples

Patient samples were obtained from archived specimens collected at the University Hospital Düsseldorf as part of the routine diagnostic. The study was approved by the Ethics Committee of the Medical Faculty, Heinrich Heine University Düsseldorf (approval number: 2024–3047). The committee granted a waiver of informed consent for the use of diagnostic leftover samples. All procedures were conducted in accordance with institutional guidelines and the Declaration of Helsinki. Wastewater samples were collected from the Düsseldorf-Süd wastewater treatment plant as part of the German national wastewater surveillance program for epidemiological assessment (AMELAG). All experiments and procedures were performed in accordance with all relevant guidelines and regulations.

### Cell culture and infection procedure

Vero cells (ATCC-CCL-81 obtained from LGC Standards) were grown in culture medium (Dulbecco’s modified essential medium (DMEM) with 1% penicillin and streptomycin (Gibco, 100 U/mL penicillin and 100 µg/mL streptomycin) and 2% fetal calf serum (FCS, PAN Biotech). Cells were maintained at 37 °C in a humidified atmosphere with 5% CO₂^34^. For infection, 7 × 10⁴ Vero cells per well were seeded into 24-well plates. The following day, nasopharyngeal swab samples were incubated for 10 min in 2 mL DMEM at room temperature. Cell culture medium was aspirated, and 100 µL of, nasopharyngeal swab suspension was added directly to each well for inoculation. Wells were then topped up with 200 µL infection medium (culture medium containing 1% trypsin) and centrifuged at 500 g for 30 min at 37 °C. Plates were subsequently incubated at 37 °C, 5% CO₂ on a tilting shaker for 1 h. After incubation, the inoculum was removed and replaced with 1 mL of fresh infection medium. Plates were then incubated at 37 °C, 5% CO₂ for 2–3 days before microscopic inspection for virus-induced cytopathic effects. Supernatant from wells showing cytopathic effects was transferred to T75 flasks seeded with 2 × 10⁶ Vero cells the previous day. Flasks were incubated at 37 °C in a humidified atmosphere with 5% CO₂ for 2–3 days before microscopic inspection for virus-induced cytopathic effects. The supernatant was then harvested, centrifuged at 2,000 × g for 10 min, and stored at − 80 °C.

### Primer design

For primer design, all available 3,403 full-length, high-quality RSV isolates from GISAID^35^ (download 19.08.2022) were aligned using MAFFT and a neighbor-joining phylogenetic tree was calculated using the Tamura-Nei distance model. The tree was reduced to 100 genetically diverse leaves using Treemmer^36^ with default parameter. RSV-A isolates were used to design RSV-A-specific tiling primers using PrimalScheme v.1.3.2 (https://primalscheme.com/) for amplification of PCR fragments with a length of approximately 500 bp. These primers were then aligned to RSV-B isolates, and in cases where the RSV-A-specific primers did not perfectly match, additional RSV-B-specific primers were designed manually. In total, we ended up with 78 RSV-A/B specific primers and additional 48 RSV-B specific primers, generating 39 overlapping amplicons. The primer sequences and the exact genomic positions and lengths of all amplicons are listed in Suppl. Tables 1 and visualized in Suppl. Figure 2. The BED files used in the RSVTyper bioconda algorithm with the primer locations and orientation can also be found on GitHub (https://github.com/DiltheyLab/RSVTyper/tree/main/rsv_typer).

### Sample preparation and nanopore sequencing

Total nucleic acids were extracted from 300 µL cell culture supernatant or nasopharyngeal swabs and eluted in 60 µL, using the Maxwell RSC Blood DNA Kit on Maxwell^®^ RSC 48 Instrument (Promega) following the manufacturer’s instructions. Total nucleic acids from wastewater samples were obtained using the Promega Enviro wastewater TNA extraction kit according to the manufacturer. Virus amplification and sequencing were performed as described in^17,37^ using the reagents from the NEBNext^®^ ARTIC SARS-CoV-2 Companion Kit (New England BioLabs). In brief, RNA was reverse transcribed in vitro with the LunaScript^®^ RT SuperMix Kit (New England BioLabs). Eight microliter RNA were mixed with 2 µL LunaScript RT SuperMix and RNA was reverse transcribed with the following conditions: 2 min at 25 °C, 20 min 55 °C, 1 min at 95 °C and hold at 4 °C. A multiplex tiling PCR was performed with RSV-specific primers divided in two pools (Suppl. Table 1), using the Q5^®^ Hot Start High-Fidelity 2X Master Mix (New England BioLabs). Pool 1 contains 65 primers and pool 2 61 primers (10µM/primer). Per reaction, 6.5 µl Q5^®^ Hot Start High Fidelity 2x Master Mix, 1.2 µL Primer-Pool (final concentration of each primer in the reaction 0.015 µM) and 4.8 µL cDNA were mixed. PCR conditions were 30 s at 98 °C, following 35 cycles of 15 s denaturing at 98 °C and 10 min annealing at 60 °C and storage at 10 °C. For DNA concentration measurement 1 µL DNA was mixed with 199 µL Quant-iT dsDNA HS Assay (Thermo Scientific) solution and fluorescence was measured on a Tristar 3 multimode plate reader (Berthold). For successfully amplified samples, defined as having a DNA concentration above 20 ng/µL, 11.5 µL of each pool were combined and purified using equal amounts of AMPure XP beads (Beckman Coulter) according to the manufacturer. Purified DNA, eluted in 25 µL nuclease-free water, was prepared for sequencing with Oxford Nanopore Technologies (ONT), using the SQK-NBD114.96 kit according to the manufacturer. In detail, for end repair 12.5 µL of purified DNA was mixed with 1.75 µL Ultra II End-prep reaction buffer and 0.75 µL Ultra II End- prep enzyme mix and incubated in a thermal cycler for 5 min at 20 °C followed by 5 min at 65 °C. Barcodes were ligated by adding 1.25 µL native ONT barcode, 0.75 µL end-prepped DNA, 3 µL nuclease-free water and 5 µL Blunt/TA Ligase Master Mix per sample and incubated for 20 min at room temperature. The reaction was stopped by adding 1 µL EDTA to each sample. The number of samples pooled depended on the type of flow cell used. Up to 48 samples were pooled on a MinION flow cell, and up to 96 samples on a PromethION flow cell. After barcoding, all samples were combined and purified using 0.4x volume of AMPure XP beads. The beads were washed twice with 700 µL 80% ethanol and the final DNA-library was eluted in 35 µL nuclease free water. For adapter ligation 30 µL of pooled, barcoded, purified sample, 5 µL of native adapter, 10 µL NEBNext Quick Ligation Reaction Buffer (5X) and 5 µL Quick T4 DNA Ligase were mixed and incubated for 20 min at room temperature. Final library was washed using 20 µL AMPure beads. The beads were washed twice with 125 µL short fragment buffer (ONT). The final library was eluted in 15 µL (32 µL for PromethION) elution buffer and 15 µL (32 µL for PromethION) of the library was mixed with 37.5 µL Sequencing Buffer (100 µL for PromethION) and 25.5 µL Library Beads (68 µL for PromethION) and loaded on a flow cell which was primed with Flow Cell Flush and Flow Cell Tether. Runtime was set to 72 h and reads were live-basecalled, discarding reads with a Q value < 10 immediately. The generated megabases, number of reads, number of reads after length-filtering, coverage, mean depth, mean depth in F gene, mean base quality and other parameters can be found in Suppl. Table 2. Using Guppy version 6.5.7, generated data were re-basecalled with the high accuracy or super accurate model and demultiplexed, enabling the parameter “--require_barcodes_both_ends”. Finally, the data were analyzed by the RSVTyper pipeline.

### Overview of the RSVTyper pipeline

The RSVTyper pipeline infers RSV genotypes and consensus sequences from nanopore sequencing data. Based on a set of reference sequences (*n* = 20), it implements the following steps. (i) Subtype detection: By counting the number of reads aligning to a region of high divergence between subtype A and subtype B, the subtype of the analyzed sample is detected. (ii) G gene duplication detection: The reads are mapped to references containing the duplication in the G gene. The number of reads supporting the absence of the duplication is counted to determine whether the sample harbors the duplication or not. (iii) Within-subtype reference selection: Based on the number of primary alignments between the reads and reference sequences with the previously called features (subtype and G gene duplication), one of them is selected to be used during the next step. (iv) Variant calling and generation of the consensus sequence: The ARTIC pipeline is executed to call variants against the previously selected reference sequence, generating a sample consensus sequence. (v) Clade assignment: “Genome-wide” clade and a “G” clade are determined from the generated consensus sequence using Nextclade.

### Generation of a 20-sequence reference panel

Due to the high diversity of RSV, analysis of the sequencing reads relative to a single reference would not lead to reliable results. Thus, a panel of 20 representative RSV reference sequences, comprising 10 sequences for each subtype was generated, by (i) curating a database of publicly available RSV genomes and by (ii) employing a clustering approach to generate 20 consensus sequences from the database.

For the creation of the database, 3,403 RSV genome sequences available on GISAID up to August 19 2022 were filtered by length and completeness, removing sequences shorter than 14,800 nt and sequences that contained ≥ 1 “N” characters, yielding a set of 3,121 sequences. NCBI RefSeq sequences for RSV-A (NC_038235.1) and RSV-B (NC_001781.1) were added. Consensus start and end positions of the filtered RSV genome sequences were identified in a multiple sequence alignment (MSA) of the 3,123 sequences, computed using MAFFT^38^. Column 389 of the MSA was the first column at which all sequences carried a non-gap character; it was thus defined as the consensus start site. The consensus end position was defined as the final position at which all sequences contained a non-gap character, located at column 15,466 of the MSA. All sequences were trimmed to the selected consensus start and end positions.

Subtype-specific clusters were generated using an iterative clustering algorithm based on distances between the trimmed RSV sequences calculated with Mash^39^. Define *S* as the set of RSV database sequences not yet assigned to any cluster and *C* as the set of clusters, initialized as the empty set. Two sequences$\:{\:x}_{1}\in\:S$, $\:{x}_{2}\in\:S$ are defined as “closely related” if and only if $\:{x}_{1}$ and $\:{x}_{2}$ are of the same subtype and if $\:mash_{d} is\tan ce\left( {x_{1} ,x_{2} } \right)lt;t$; $\:t$ is defined as 0.0062 for subtype A and as 0.0065 for subtype B. These threshold were chosen so that we could obtain 10 clusters with at least 10 sequences. The following steps are carried out to populate *C*:

1. For each element $\:x$ in $\:S$, determine the number $\:{r}_{x}$ of other elements in $\:S$ closely related to $\:x$.
2. Determine the element $\:{x}_{\:max}=\:\underset{x\in\:S}{\text{arg max}}{r}_{x}$ (that is, $\:{x}_{\:max}$ is the element of $\:S$ with the highest number of closely related elements in $\:S$).
3. Set cluster $\:k=\:\left|C\right|+1$ as the union of $\:{x}_{\:max}$ and the set of all elements in $\:S$ closely related to $\:{x}_{\:max}$.
4. Remove all members of cluster $\:k$ from $\:S$.
5. Terminate if $\:\left|S\right|=1$ or if $\:\left|k\right|$ is 1; otherwise, go to Step 1.

In the final step, 20 reference sequences were generated by computing consensus sequences from the first 10 clusters of each subtype, selecting the nucleotide that was found in the majority of the untrimmed sequences for each position in the MSA or, in case of a draw, the nucleotide was selected randomly using the Python method “random.choice()”. If the majority of sequences carried a gap character, no character was added to the consensus sequence. Leading gaps were not considered in the consensus sequence generation. Sequences belonging to each cluster are listed in Suppl. Table 4.

We wanted to ensure completeness of each reference sequence with respect to the NCBI RefSeq sequences for RSV-A and RSV-B, using the beginning (position 44) and the end (position 15818) of the NCBI RefSeq sequences in the MSA as reference points. Five subtype A and six subtype B reference sequences were missing nucleotides at the beginning and/or the end of their sequence due to incompleteness of the sequences used in consensus sequence generation. Therefore, a subset of sequences containing a complete sequence, i.e. a non-gap character at MSA positions 44 and 15,818, was extracted. For each cluster with an incomplete consensus sequence, the sequence from the subset of complete sequences with the lowest average distance to all members of the cluster was determined. The missing nucleotides were then filled up using the determined sequence. Reference sequences, which contained nucleotides outside of the NCBI RefSeq references were trimmed to fit the RefSeq references.

### Subtype detection

When sequencing samples that carry both, RSV-A and RSV-B, which could occur naturally or due to contamination, the variant caller will call and try to implement variants from both subtypes into the consensus sequence. To prevent the generation of an artificial chimeric RSV sequence consisting of both subtypes, a subtype detection step is included in the pipeline. For subtype detection, the basecalled reads are aligned against a file containing the 20 reference sequences using minimap2^40^ with the parameters “-ax map-ont”, discarding all secondary alignments. The number of reads aligning to a region of high sequence diversity between A and B subtypes (approximate coordinates 3,500–5,500; see Suppl.Table 5 for the MSA coordinates) is determined using samtools^41^. max_A and max_B are defined as the maximum number of reads aligned to the target region within any one of the 10 reference sequences of RSV-A and RSV-B, respectively. If max_A or max_B exceeds the other by a factor of at least 5, the corresponding subtype is called. Else, no subtype is determined and the pipeline is aborted.

### G gene duplication detection

RSVTyper first determines whether the sequenced sample contains the G gene duplication. This prevents errors in consensus sequence generation that can occur when data from an isolate with the duplication is analyzed using a reference sequence without it, or vice versa. Reads are length-filtered to 350-900 nt using artic guppyplex with “--skip-quality-check” and mapped to a file containing all reference sequences of the respective subtype (determined during the previous step) that harbor the G gene duplication. RSVTyper then determines the proportion of primary read alignments consistent with the absence of the G gene duplication in the sequenced isolate, defined as the proportion of primary read alignments that carry contiguous deletions of > = 60 nt (RSV-A) or > = 50 nt (RSV-B) in size in the region of +/- 50 bases around the genomic start and stop locations of the G gene duplication (see Suppl. Table 6 for exact coordinates). If the proportion of primary read alignments consistent with the absence of the G gene duplication is > = 70%, the sequenced isolate is assumed to not carry the G gene duplication and only references without the duplication are used in downstream analysis. Otherwise, only references with the duplication are used.

### Within-subtype reference selection

The reads are aligned against a file containing all reference sequences of the previously called subtype with or without the duplication using minimap2 and the number of primary alignments is counted for each reference. The reference sequence with the highest number of primary alignments is used for the next step.

### Variant calling and generation of a consensus sequence with the ARTIC pipeline

To call variants and generate a consensus sequence from the reads of the analyzed sample, the ARTIC minion pipeline (https://github.com/artic-network/fieldbioinformatics) is executed with the parameters “--no-longshot”, “--medaka”, “--normalise 100000” and the appropriate medaka model (r1041_e82_400bps_hac_variant_v4.2.0 for the MinION runs and r1041_e82_400bps_sup_variant_v4.3.0 for the PromethION runs). The artic pipeline comprises the following steps: First, chimeric reads are filtered out based on the minimum and maximum amplicon lengths. This step is already done during G gene duplication detection. Second, the filtered reads are aligned to the previously chosen reference sequence with minimap2 and the alignments are processed, which includes the assignment of read alignments to derived amplicons and trimming of primer sequences, excluding them in the variant calling. Finally, the experimental medaka pipeline is executed to call variants and generate a consensus sequence. In alignment with the ARTIC community standard complete genomes were defined > 90% genome coverage with a minimum depth of 20 reads^18,19,22,42^.

### Clade assignment with nextclade

“Genome-wide” and “G” clade data are inferred using Nextclade^25^, based on the consensus and the previously called sample subtype. For the “genome-wide” clade assignment the whole genome is considered while the “G” clade assignment only considers the G gene^43^.

### Hardware requirements

The pipeline runs on Linux and requires a GPU with 11 GB memory, 4 CPU cores and 4 GB RAM. Software dependencies are specified in the Github Repository (https://github.com/DiltheyLab/RSVTyper).

### Wastewater sample analysis

RSV variant abundance in wastewater samples was detected using Freyja v1.5.1^27^ with RSV-A and RSV-B barcodes (https://github.com/andersen-lab/Freyja-barcodes/). Only variants with an abundance > 0.05 in at least one sample were included.

### Construction of phylogenetic trees

RSV-A and RSV-B sequences with a genome coverage > 90% were aligned separately using MAFFT v7.520. Neighbor-joining trees were computed with MEGA v11.03.13^44^, using the Tamura-Nei model., and visualized using iTOL v7^45^. For consensus trees phylogenetic analysis a tree based on the complete RSV sequence, with references from Genebank, was calculated with the Mr. Bayes plugin 26 using the ngphylogeny-pipeline (https://ngphylogeny.fr/) with default parameter.

### Recombination analysis

Recombination analysis were performed by Pairwise Homoplasy Index-Test (Phi)^46^ implemented in the SimPlot + + package^47^ using a window size of 100 and 1000 permutations.

## Supplementary Information

Below is the link to the electronic supplementary material.

Supplementary Material 1

Supplementary Material 2

Supplementary Material 3

Supplementary Material 4

Supplementary Material 5

Supplementary Material 6

Supplementary Material 7

Supplementary Material 8

## Data Availability

The viral genome sequences are provided in Suppl. Material1 (FASTA format, without metadata). The complete datasets, including associated metadata, are available through GISAID [EPI_ISL_20060811- EPI_ISL_20061029] https://doi.org/10.55876/gis8.250822yc.
