# Supplementary material for: Whole-genome nanopore sequencing and automatic downstream analysis of respiratory syncytial virus using RSVTyper
Source: Sci Rep. 2025 Oct 31;15:38101. doi: 10.1038/s41598-025-20371-5 (PMC12578865; doi:10.1038/s41598-025-20371-5)
Supplement: Supplementary file 8 — Supplementary Material 8 [file 41598_2025_20371_MOESM8_ESM.docx]

**RSVTyper: A multiplex tiling approach for amplification, sequencing and downstream analysis of Respiratory Syncytial Virus**

Duyen Bao Le, Inga Tometten, Nadine Lübke, Martha Paluschinski, Anna-Kathrin Schupp, Lutz Ehlkes, Pascal Kreuzer, Nicole Zacharias, Jörg Timm, Alexander Dilthey, Andreas Walker

**Suppl. Table 1**

RSV-Primer developed in this study and position and size of the amplicons.

**Suppl. Table 2**

Output of the RSVTyper for all Isolates sequenced in this study, sequencing statistics and GISAID accession numbers.

**Suppl. Table 3.**

The Nextclade output for all samples. Data is sorted in sheets according to material and RSV subtype.

**Suppl. Table 4.**

Final reference sequences generated during the reference construction process, together with the accession numbers of all samples within the clusters used for final reference generation.

**Suppl. Table 5.**

Target regions used for subtype detection in MSA coordinates

**Suppl. Table 6.**

Coordinates used for the G-Gene duplication detection

**Suppl. Material 1**

Consensus sequences generated by the RSVTyper.

**Suppl. Figure 1**

RSV-B variant abundances in wastewater from RSV season 2023/2024. RSV-B variants detected in wastewater samples from 12 different timepoints are compared to RSV-B (n = 4) variants detected in patients from the University Hospital Düsseldorf, sampled within the same time frame, respectively.

**Suppl. Figure 2**

Schematic illustration of the primer binding sites and amplicon position.

**Suppl. Figure 3**

Recombination analysis were performed with SimPlot++ [1] using a window size of 200, step size of 20 and the Jukes-Cantor genetic model. The RSV-B consensus sequence of the 2022-2024 isolates was used as query reference.

1. Samson, S., E. Lord, and V. Makarenkov, *SimPlot++: a Python application for representing sequence similarity and detecting recombination.* Bioinformatics, 2022. **38**(11): p. 3118-3120.
